# Transcriptome analysis unraveled potential mechanisms of resistance to *Haemonchus contortus* infection in Merino sheep populations bred for parasite resistance

**DOI:** 10.1186/s13567-019-0622-6

**Published:** 2019-01-24

**Authors:** Runfeng Zhang, Fang Liu, Peter Hunt, Congjun Li, Lichun Zhang, Aaron Ingham, Robert W. Li

**Affiliations:** 1 College of Life Science, Hubei Normal University, Huangshi, Hubei China; 2 College of Food Science and Engineering, Ocean University of China, Qingdao, China; 3 CSIRO Agriculture and Food, Armidale, NSW Australia; 4 United States Department of Agriculture, Agriculture Research Service, Animal Genomics and Improvement Laboratory, Beltsville, MD USA; 5 Branch of Husbandry, Jilin Academy of Agricultural Science, Gongzhuling, Jilin China; 6 CSIRO Agriculture and Food, St Lucia, Queensland, Australia

## Abstract

*Haemonchus contortus* is one of the most pathogenic gastrointestinal nematodes in small ruminants. To understand molecular mechanisms underlying host resistance to this parasite, we used RNA-sequencing technology to compare the transcriptomic response of the abomasal tissue, the site of the host-parasite interaction, of Merino sheep bred to be either genetically resistant or susceptible to *H. contortus* infection. Two different selection flocks, the *Haemonchus* selection flock (HSF) and the *Trichostrongylus* selection flock (TSF), and each contains a resistant and susceptible line, were studied. The TSF flock was seemingly more responsive to both primary and repeated infections than HSF. A total of 127 and 726 genes displayed a significant difference in abundance between resistant and susceptible animals in response to a primary infection in HSF and TSF, respectively. Among them, 38 genes were significantly affected by infection in both flocks. Gene ontology (GO) enrichment of the differentially expressed genes identified in this study predicted the likely involvement of extracellular exosomes in the immune response to *H. contortus* infection. While the resistant lines in HSF and TSF relied on different mechanisms for the development of host resistance, adhesion and diapedesis of both agranulocytes and granulocytes, coagulation and complement cascades, and multiple pathways related to tissue repair likely played critical roles in the process. Our results offered a quantitative snapshot of changes in the host transcriptome induced by *H. contortus* infection and provided novel insights into molecular mechanisms of host resistance.

**Electronic supplementary material:**

The online version of this article (10.1186/s13567-019-0622-6) contains supplementary material, which is available to authorized users.

## Introduction

Gastrointestinal nematodes (GIN) represent a major health issue for livestock production systems worldwide [1]. GIN infection causes serious losses to farmers, both in impaired production and in control with anthelmintics. Anthelmintics are rapidly becoming ineffective against economically important GIN, due to the increasing incidence of anthelmintic resistance [2]. Moreover, current reliance on anthelmintics has resulted in public concerns for animal welfare and the contaminating residues in animal products [3]. Together, these factors have driven the development of effective and sustainable control strategies, including the application of novel vaccines that can be used to increase flock resistance and the development of genetically resistant populations [4, 5].

Immunological protection against GIN infection is associated with T-helper (Th) responses as well as tissue repair systems. Compared with susceptible animals, which usually evoke a response that shows characteristics of a Th1 response, resistant animals typically produce a dominant, polarized Th2 immune response [6, 7]. Therefore, a hallmark of immunity to GIN is a strong Th2 immune response [8, 9], characterized by the production of cytokines interleukin 4 (IL4), IL5, and IL13 [10]. In sheep resistant to GIN, the characteristics of a Th2 host response include the production of elevated levels of parasite-specific immunoglobulin A (IgA), IgE and IgG_1_ antibodies [11], eosinophilia [12], mucosal mastocytosis, and goblet cell hyperplasia [13]. However, the molecular mechanism of host resistance to *H. contortus* has yet to be fully understood.

In this study, we took advantage of the availability of a valuable sheep model that includes two resource flocks developed by selective breeding for genetic resistance or susceptibility to *H. contortus* [14] and *Trichostrongylus colubriformis* [15] infections. We used a high-throughput RNA sequencing (RNA-seq) approach in an attempt to identify features in the host transcriptome using both sets of flocks in response to an experimental *H. contortus* challenge. As such, the work reported here is considered as a companion to and complementary with previous studies [9, 16, 17] in which the candidate gene approach, microarray technology, and proteomic analysis were carried out using the same model. While these prior studies have led to valuable findings and resulted in some working hypotheses for future research, they are largely based on either a limited number of candidate genes or cross-species comparisons (ovine to bovine for microarrays and ovine to human for proteomics). A holistic analysis of the abomasal transcriptome in these resource populations using high-throughput RNA-seq technology and bioinformatics tools, especially on novel transcriptome features, such as transcript isoforms and alternative splicing, will facilitate our understanding of molecular mechanisms of resistance to *H. contortus* infection.

## Materials and methods

### Animals and parasitology

All lambs were obtained from two resource flocks of Merino sheep, the *Haemonchus* selection flock (HSF) and the *Trichostrongylus* selection flock (TSF), developed by selection and assortative mating for exploring the genetic basis of host resistance to nematodes [16, 18]. Each selection flock contains a resistant (R) line and a susceptible (S) line. The lambs in this study were reared in an animal housing facility on wooden slats from birth. Prior to the commencement of the challenge experiment by *H. contortus*, lambs from each line were divided into “innate” (I) and “acquired” (A) experimental infection groups. There were between six and seven lambs in each of these groups (Table 1). Lambs in the innate groups (I) received a primary challenge, whereas those in the acquired groups (A) received tertiary challenges. The acquired groups were challenged twice at 16 and 27 weeks of age, respectively, with 5000 *H. contortus* L3 larvae each (the Kirby strain). The infections were allowed to last for 6 weeks before being terminated using a single dose of 8.25 mg/kg levamisole hydrochloride treatment. At 37 weeks of age, a final infection (3^rd^) for the acquired groups and a primary (1^st^) infection for the innate groups were administrated with 10 000 *H. contortus* L3 larvae. All animals were euthanized on the third day after this infection. All animal procedures were carried out according to the protocols approved by the New South Wales (NSW) Department of Primary Industries, Director General’s Animal Care and Ethics Committee; and Australian Animal Welfare Standards and Guidelines for sheep were strictly followed (Animal Protocol# 03/75, 04/49 and 05/58).

**Table 1:** **Groups of sheep used during** ***Haemonchus contortus*** **challenge trials**

Selection flock	Line	Group	Group symbol
	Resistant (R)/susceptible (S)	Innate (I)/acquired (A)	(# Animals, *N*)
HSF	R	I	HRI (6)
		A	HRA (7)
	S	I	HSI (6)
		A	HSA (6)
TSF	R	I	TRI (6)
		A	TRA (7)
	S	I	TSI (6)
		A	TSA (6)

### RNA extraction and sequencing using RNA-seq technology

Abomasal tissues were scraped and snap frozen in liquid nitrogen and then stored at −80 °C until use. Total RNA samples from the abomasal scrapings were isolated using Trizol (Invitrogen, Carlsbad, CA, USA) followed by DNase digestion and Qiagen RNeasy column purification (Qiagen, Valencia, CA, USA). RNA concentrations were determined using a NanoDrop ND-1000 spectrophotometer (Thermo Scientific, Wilmington, MA, USA), and the RNA integrity was verified using an Agilent BioAnalyzer 2100 (Agilent, Palo Alto, CA, USA) with a RNA integrity number (RIN) value > 7.5. High-quality RNA was processed using an Illumina TruSeq RNA sample prep kit (Illumina, San Diego, CA, USA), following the manufacturer’s instructions. The concentrations of individual RNA-seq libraries were verified and then pooled according to their respective index barcodes. Pooled RNA-seq libraries were sequenced as paired-end reads at 51 bp/sequence using an Illumina HiSeq 2000 sequencer (Illumina), as previously described [19, 20]. The metadata and raw sequence files were deposited in the National Center of Biotechnology Information (NCBI) Sequence Read Archive (SRA access number PRJNA445172).

### Data analysis and bioinformatics

Raw sequence reads (mean ± SD = 65 699 458 ± 18 187 533 per sample, *N* = 50) were first checked using FastQC (Babraham Institute, Cambridge, UK). Low-quality reads were discarded; and low-quality nucleotides of each raw sequence read were trimmed using Trimmomatic [21] with default parameters. The resultant quality reads (mean reads = 57 921 126 per sample) after cleansing were mapped to the sheep reference genome Oar_v3.1 using TopHat2 (version 2.1.1) [22]. TopHat2 was run with the ‘‘–no coverage search’’ option and transcriptome indices were provided via the ‘‘–transcriptome-index’’ option. All other parameters were default settings. Transcripts were assembled and quantified using Cufflinks (version 2.2.0) [23], which was run with “-u” (multi-read correction) and “-b” (bias-correction) options. Transcript assemblies generated by Cufflinks were merged into a single transcriptome annotation using Cuffmerge (version 2.1.1) [23]. The reference annotation file and genomic sequence were provided to Cuffmerge via the “-g” and “-s” options, respectively. Differential expression analysis was performed using Cuffdiff (version 2.2.1) with the ‘‘-u’’ (multi-read correction) and ‘‘-b’’ (bias-correction) options. Differentially expressed genes (DEG) were extracted with the aid of the cummeRbund (R package), using a false discovery rate (FDR) < 0.05 as a cut-off and including an additional fold change filter (> 1.5-fold). The comparison was made independently between resistant and susceptible lines in each flock in response to different infection protocols. In addition, the raw data were also analyzed independently using the STAR-RSEM-EdgeR pipeline as previously described [24] and the CLC Genomics Workbench (Qiagen). Raw reads were mapped to both the sheep genome assemblies Oar_v3.1 and Oar_v4.0 using the ultrafast STAR aligner [25] and the CLC Genomics Workbench, respectively. DEG were further analyzed with Ingenuity Pathways Analysis (IPA) software (Qiagen), essentially as described previously [26]. Gene ontology (GO) and the Kyoto Encyclopedia of Genes and Genomes (KEGG) assignments were analyzed with Database for Annotation, Visualization and Integrated Discovery (DAVID v6.8) [27]. In addition, gene enrichment analysis was performed using ShinyGO v0.50. Principal component analysis (PCA) was conducted using Primer v7.0 (PRIMER-E, Plymouth, UK).

### Validation by real-time reverse transcription polymerase chain reaction (qRT-PCR)

The relative expression of 12 genes (see Additional file 1 for their primer sequences) was determined by qRT-PCR, as previously described [19]. Ovine ribosomal protein L19 gene (RPL19), whose expression remained stable among the experimental samples, was used as an endogenous reference gene for all reactions. cDNA was synthesized from total RNA using an iScript cDNA synthesis kit (BIO-RAD, Hercules, CA, USA), according to the manufacturer’s instructions. All qRT-PCR reactions were carried out in a Mic Real-Time PCR Cycler (Bio Molecular Systems, Australia) and analyzed with micPCR v2.2 software (Bio Molecular Systems, Upper Coomera, QLD, Australia). The reactions were run in triplicate in a total volume of 20 μL containing the following: 2 μL of cDNA (200 ng), 0.5 μL of each primer (forward and reverse, 20 nM each), 10 μL of 2 × SensiFAST No-ROX Mix (Bioline, MA, USA) and 7 μL of nuclease-free water. The amplification reactions were subjected to an initial denaturation at 95 °C for 5 min, followed by 40 cycles of 95 °C for 10 s, 60 °C for 30 s, and 72 °C for 20 s. Melting curves were obtained from 60 to 95 °C. Relative gene expression data was calculated using the 2^−ΔΔCT^ method.

## Results

### Differences in the abomasal transcriptome of two selection flocks in response to experimental *H. contortus* infections

**Figure 1:**
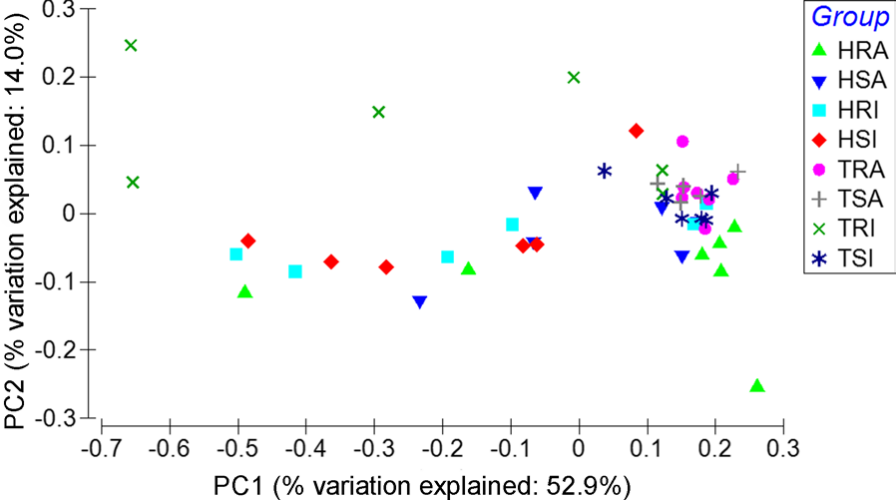
**Principal component analysis (PCA) based on normalized raw counts.** H: *Haemonchus* selection flock (HSF); T: *Trichostrongylus* selection flock (TSF); R: resistant line; S: susceptible line; I: innate infection protocol (primary infection); A: acquired infection protocol (repeated infection).

**Figure 2:**
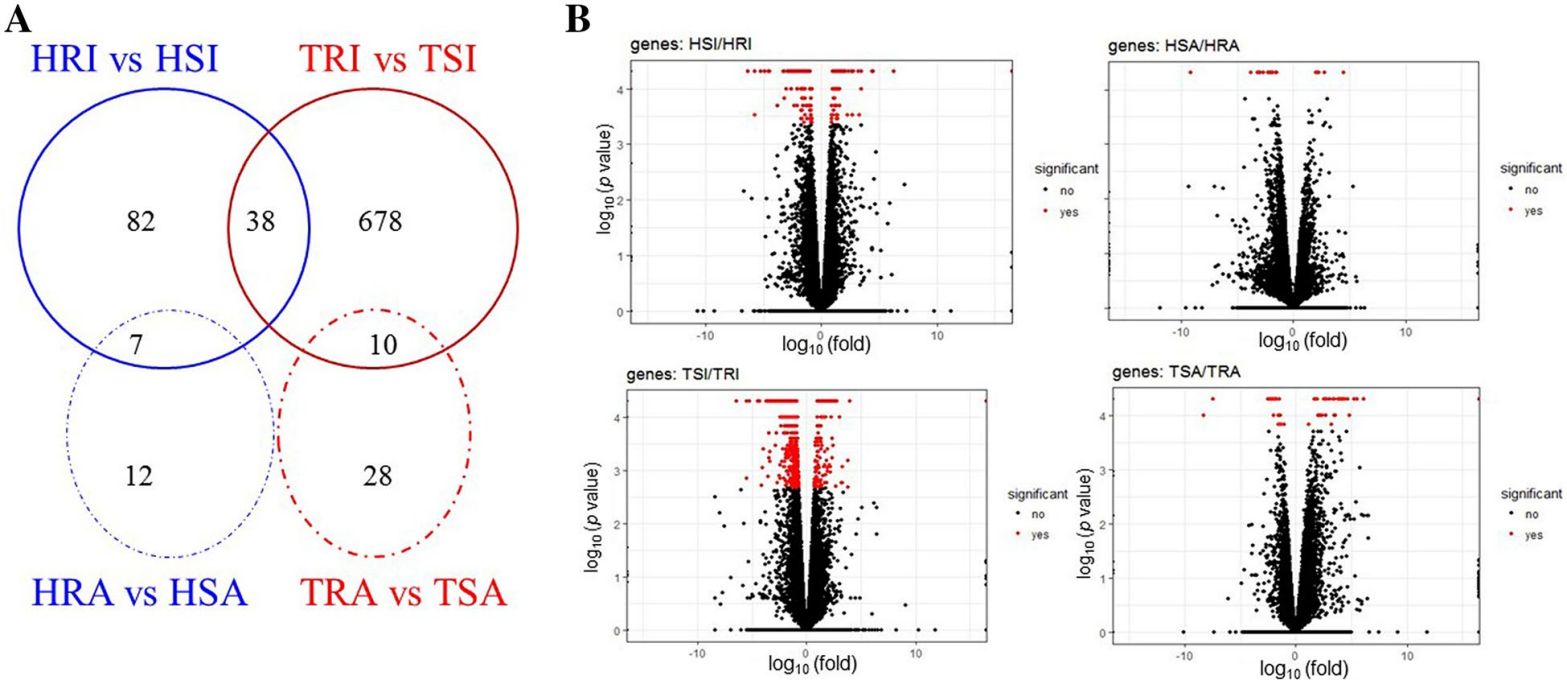
**Venn diagrams and Volcano plots. A** Venn diagram showing the number of genes with significant differences in relative abundance induced by infection point in two sheep flocks when comparing the resistant lines with their respective susceptible counterparts at a false discovery rate (FDR) cutoff < 0.05. **B** Volcano plots. The red color indicates genes detected as differentially expressed between the resistant groups and susceptible groups at FDR < 0.05. H: HSF flock; T: TSF flock; R: resistant line; S: susceptible line; I: innate infection protocol (primary infection); A: acquired infection protocol (repeated infection).

In this study, approximately 76.40% of raw reads (± 4.05%, SD) were uniquely mapped to the ovine genome assembly (Oar_v3.1) using TopHat2. PCA analysis shows that the separation between the two flocks, between the two lines (Additional file 2), or among the various experimental groups (Figure 1) was indistinguishable, suggesting that neither long-term selective breeding nor different experimental *H. contortus* infection protocols had a profound effect on the overall abomasal transcriptome structure and composition. Nevertheless, the extent of the changes in the abomasal transcriptome of different lines in different flocks in response to differential infection protocols differed. The numbers of DEG identified between various groups are shown in Figure 2. We identified 127 and 726 DEGs in the innate infection groups between the R and S lines within the HSF and TSF flocks, respectively, while the number of DEG identified in the acquired groups was 19 and 378 between the R and S lines within the HSF and TSF flocks, respectively. It appeared that greater numbers of DEG were identified in the groups using the innate infection protocols than those using the acquired protocol (Additional files 3, 4, 5, 6), suggesting the abomasal transcriptome of the lambs may be more responsive to a primary infection, in both flocks. Moreover, the abomasal transcriptome of the animals in the TSF flock was seemingly more responsive than those in the HSF flock, regardless of the infection protocols used. The expression of 38 genes was significantly (FDR < 0.05) impacted by *H. contortus* infection in both flocks when R lines were compared to their respective S lines in the innate infection (Table 2). These DEG likely represented a set of early response genes to *H. contortus* primary infections. Among them, the expression of 9 genes, such as heat shock protein family A (Hsp70) member 6 (HSPA6), lectin, galactoside-binding, soluble, 15 (LGALS15), and complement factor I (CFI), were significantly enhanced by infection in both R lines when compared to their respective S lines.

**Table 2:** **38 genes displayed significant differences (false discovery rate FDR < 0.05) in the relative abundance in response to a primary infection in both sheep** ***Haemonchus*** **selection (HSF) and** ***Trichostrongylus*** **selection (TSF) flocks selected for parasite resistance**

Gene_id	Symbol	HRI vs HSI		TRI vs TSI	
		Fold	FDR	Fold	FDR
ENSOARG00000016787	BST2	0.27	0.0101	3.72	0.0192
ENSOARG00000012075	CDON	0.40	0.0101	2.20	0.0125
ENSOARG00000005291	CFI	3.62	0.0101	2.65	0.0026
ENSOARG00000010444	CHRDL2	0.46	0.0342	4.14	0.0026
ENSOARG00000000237	DAAM2	0.47	0.0342	2.13	0.0046
ENSOARG00000012400	EPHA2	1.87	0.0383	2.01	0.0081
ENSOARG00000007427	EPSTI1	0.46	0.0288	6.09	0.0026
ENSOARG00000006366	FCGBP	2.02	0.0170	2.10	0.0026
ENSOARG00000016664	FGG	5.94	0.0101	0.24	0.0026
ENSOARG00000001783	FOS	1.78	0.0170	2.67	0.0026
ENSOARG00000007839	GEM	0.47	0.0170	2.41	0.0026
ENSOARG00000019963	GJA1	0.55	0.0427	2.30	0.0026
ENSOARG00000015775	GPT2	1.84	0.0342	0.52	0.0046
ENSOARG00000014902	GSTA1	3.03	0.0101	0.35	0.0026
ENSOARG00000015352	HAND2	0.38	0.0101	3.02	0.0026
ENSOARG00000007452	HSPA6	9.94	0.0101	4.75	0.0046
ENSOARG00000015177	IFIT1	0.20	0.0101	11.58	0.0026
ENSOARG00000015169	IFIT2	0.25	0.0101	7.11	0.0026
ENSOARG00000003270	KCNMB1	0.49	0.0383	2.32	0.0026
ENSOARG00000019088	LGALS15	6.98	0.0101	9.94	0.0026
ENSOARG00000003052	MAOA	0.41	0.0101	2.23	0.0026
ENSOARG00000011577	PDIA2	2.83	0.0101	0.26	0.0026
ENSOARG00000019839	PDZRN4	0.45	0.0101	3.45	0.0026
ENSOARG00000008451	PRR15	2.01	0.0101	2.56	0.0026
ENSOARG00000021142	RHOJ	0.51	0.0170	2.00	0.0046
ENSOARG00000020495	RTP4	0.27	0.0101	8.32	0.0026
ENSOARG00000013062	SETD7	0.54	0.0383	2.16	0.0026
ENSOARG00000020750	SMYD1	0.47	0.0170	3.07	0.0026
ENSOARG00000004567	STON1	0.55	0.0471	1.82	0.0444
ENSOARG00000012923	TACR1	0.39	0.0101	3.38	0.0026
ENSOARG00000020741	TNFSF10	0.40	0.0101	1.81	0.0259
ENSOARG00000006096	WFDC2	2.28	0.0101	2.43	0.0026
ENSOARG00000003275	WFIKKN2	0.41	0.0170	2.50	0.0125
ENSOARG00000002631		2.55	0.0101	2.25	0.0046
ENSOARG00000002875		0.40	0.0170	3.30	0.0026
ENSOARG00000002881		0.16	0.0101	6.63	0.0026
ENSOARG00000009474		2.10	0.0288	0.35	0.0026
ENSOARG00000018075		0.24	0.0101	4.34	0.0026

Three and 17 genes significantly impacted by infection in response to the innate protocol between the R and S lines are related to extracellular matrix (ECM) within the HSF and TSF flocks, respectively (Additional files 3 and 4). Of note, all of them were significantly upregulated in the R lines when compared to their respective S lines. TFF3, involved in wound healing, mucosal protection, cell proliferation and cell migration, was strongly upregulated (21.54-fold) by infection in the R line of the HSF flock when compared to its S counterparts. Moreover, the mRNA expression of several cytokine receptors and chemokines were significantly impacted by infection (FDR < 0.05). The transcripts of IL15 receptor subunit alpha (IL15RA) and IL22 receptor subunit alpha 1 (IL22RA1) were 2.50- and 3.50-fold higher in R animals than S animals in response to the innate challenge protocol in the TSF flock (Additional file 3). Notably, the abundance of seven chemokines, such as C–C motif chemokine ligand 14 (CCL14), CCL20, CCL25, CCL5, C–X–C motif chemokine ligand 10 (CXCL10), CXCL14, and atypical chemokine receptor 3 (ACKR3), was significantly higher by infection in the R line of the TSF flock (Additional file 3). Similarly, CCL19, CCL22, and CXCL13 were significantly upregulated by a primary infection in the R line of the HSF flock when compared to its S line counterparts (Additional file 4).

Of note, greater than 20% of the genes significantly impacted by infection in both flocks are extracellular exosome-related (Additional file 7). The expression of these extracellular exosome-related genes was predominantly enhanced in the R lines compared to their respective S lines. At least 121 genes were significantly upregulated by infection in the R line of the TSF flock, such as adiponectin, C1Q and collagen domain containing (AdipoQ), annexin A1 (ANXA1) and ANXA 3, various complement related genes (CFI, C1QA, C1QB, C1QC, and C1S), and integrin subunit alpha 1 (ITGA1) and ITGA3. On the other hand, 24 genes were significantly upregulated by infection in the R line of the HSF flock, such as CD19, CD22, CD79B, two complement-related genes CFI and CR2 (complement C3d receptor 2), FGG, and FGA (fibrinogen alpha chain). Nevertheless, the infection was also able to repress extracellular exosome-related genes, such as complement C7 (C7) in the R line of the HSF flock, and fibroblast growth factor 9 (FGF9), cadherin 16 (CDH16), and FGG, in the R line of the TSF flock.

### Gene ontology (GO), pathways, and molecular networks related to host resistance

Gene enrichment analysis using ShinyGO identified a total of 14 GO terms shared by the two flocks in response to the innate infection protocols (FDR < 0.0001), including 5 Biological process (BP), 8 Cellular components (CC), and 1 Molecular function terms. For example, GO terms, such as Extracellular space, Fibrinogen complex, Response to external stimulus, and Signaling receptor binding, were significantly enriched in both comparisons, TRI vs TSI and HRI vs HSI. However, only 1 GO term, Extracellular space (GO:0005615), was significantly enriched in both flocks in response to the acquired infection protocol. Moreover, Inflammatory response (GO:0006954), Integrin-mediated signaling pathway (GO:0007229), Cell adhesion (GO:0007155) and cell matrix adhesion (GO:0007160) were seemingly among important biological processes involved in the development of resistance to a primary infection. In addition, the cellular component Extracellular exosome (GO:0070062) may play an important role in the development of host resistance, regardless of the flock origin and infection status (Additional file 7).

**Figure 3:**
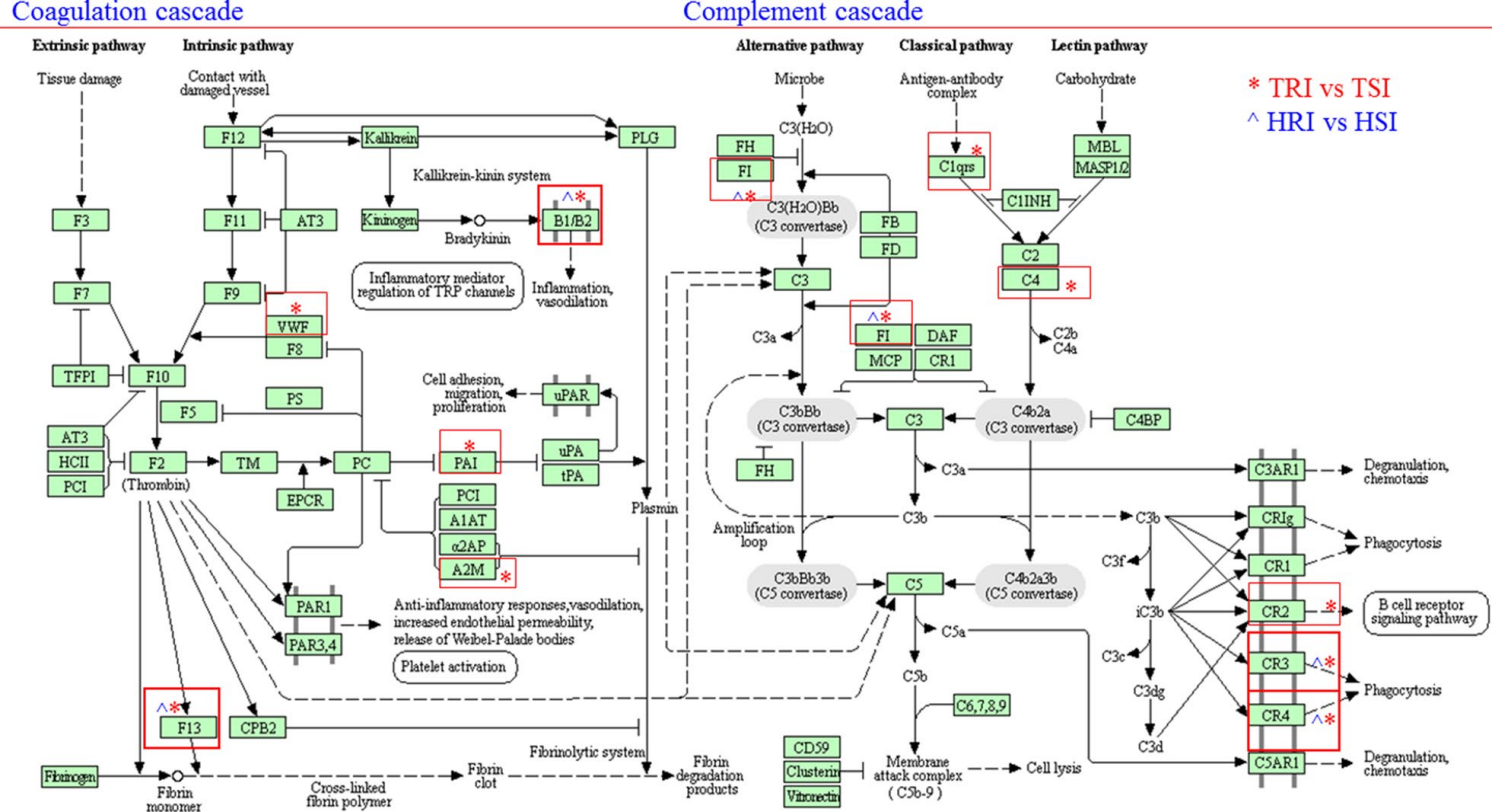
**Biological pathways significantly enriched in response to a primary infection.** The Kyoto Encyclopedia of Genes and Genomes (KEGG) pathways Complement and coagulation cascades were significantly enriched at a FDR < 10% in both HSF and TSF flocks in response to a primary *Haemonchus contortus* infection. *Denotes genes that displayed a significantly different abundance between R and S lines in the TSF flock. ^^^Denotes genes that displayed a significantly different abundance between R and S lines in the HSF flock.

A total of 20 KEGG pathways were significantly impacted in the innate immune response to infection between the R and S lines in the TSF flock (FDR < 0.1; see Additional file 8). These pathways, such as leukocyte transendothelial migration, MAPK signaling, TNF signaling, cell adhesion molecules, focal adhesion (Table 3), and ECM-receptor interaction (Table 4), were likely involved in the development of host resistance to a primary infection in TSF. On the other hand, only two pathways, Complement and coagulation cascades, and B cell receptor signaling, were significantly enriched between the R and S lines in the HSF flock. Of note, Complement and coagulation cascades (Figure 3), was also significantly enriched in the TSF flock, suggesting that this pathway likely contributed to the development of host resistance to a primary *H. contortus* infection. Similarly, IPA gene enrichment analysis identified several canonical pathways that were significantly enriched in both flocks in response to the acquired infection protocol. Among these, coagulation system was significantly enriched in both HSF and TSF flocks in response to the acquired infection protocol, in a good agreement with the DAVID results. On the other hand, Interferon signaling was enriched in the HSF flock while the pathway IL-17A signaling in gastric cells may play an important role in the TSF flock.

**Table 3:** **The genes related to gene ontology (GO) term focal adhesion significantly enriched in the** ***Trichostrongylus*** **selection flock (TSF) in response to a primary** ***Haemonchus*** **infection**

Gene_ID	Symbol	TRI_FPKM	TSI_FPKM	Fold_TRI/TSI	*P*_value	FDR
ENSOARG00000021179	ACTN1	58.98	27.48	2.15	5.00E−05	0.0026
ENSOARG00000018737	ALCAM	10.44	18	0.58	7.00E−04	0.0215
ENSOARG00000005944	ADGRE5	24.95	11.64	2.14	9.00E−04	0.0259
ENSOARG00000003112	AKAP12	7.15	3.58	2	2.00E−04	0.0081
ENSOARG00000012672	ANXA1	322.13	155.3	2.07	5.00E−05	0.0026
ENSOARG00000009566	CDH13	10.02	5.78	1.73	1.55E−03	0.0393
ENSOARG00000018621	CNN1	640.1	200.68	3.19	5.00E−05	0.0026
ENSOARG00000001337	CAV1	96.87	55.32	1.75	1.20E−03	0.0322
ENSOARG00000018809	CD44	35.67	19.63	1.82	7.50E−04	0.0229
ENSOARG00000008605	CD9	465.28	233.42	1.99	1.00E−04	0.0046
ENSOARG00000001908	CSPG4	8.76	4.53	1.93	8.00E−04	0.024
ENSOARG00000017927	CORO1C	19.16	11.58	1.65	1.95E−03	0.0459
ENSOARG00000006454	DPP4	9.24	17.31	0.53	2.50E−04	0.0097
ENSOARG00000017329	ENAH	25.61	13.47	1.9	1.75E−03	0.0427
ENSOARG00000005448	EFNB2	12.25	6.57	1.87	3.50E−04	0.0125
ENSOARG00000021064	FERMT2	34.37	18.69	1.84	3.00E−04	0.0113
ENSOARG00000005208	FLNA	168.68	71.28	2.37	8.50E−04	0.025
ENSOARG00000012724	FLNB	61.8	31.33	1.97	6.50E−04	0.0203
ENSOARG00000003372	FLNC	20	4.87	4.11	5.00E−05	0.0026
ENSOARG00000002088	GRK5	6.36	3.72	1.71	1.15E−03	0.0313
ENSOARG00000019963	GJA1	13.71	5.95	2.3	5.00E−05	0.0026
ENSOARG00000008285	ITGA1	9.86	5.47	1.8	3.00E−04	0.0113
ENSOARG00000005412	ITGA3	32.43	19.1	1.7	8.00E−04	0.024
ENSOARG00000016181	ITGA5	17.19	9.68	1.78	1.05E−03	0.0291
ENSOARG00000016024	LIMS2	50.95	26.06	1.96	5.00E−05	0.0026
ENSOARG00000019414	MMP14	26.31	13.95	1.89	1.50E−04	0.0064
ENSOARG00000009328	MISP	10.9	3.2	3.41	5.00E−05	0.0026
ENSOARG00000008298	PARVA	32.29	19.49	1.66	2.15E−03	0.0493
ENSOARG00000013106	PGM5	63.63	35.25	1.81	1.60E−03	0.0401
ENSOARG00000009699	PTK2B	15.5	9.21	1.68	1.25E−03	0.0332
ENSOARG00000019441	RHOB	40.46	21.92	1.85	1.15E−03	0.0313
ENSOARG00000009225	RND3	13.67	5.86	2.33	5.00E−05	0.0026
ENSOARG00000014369	SVIL	15.58	7.81	1.99	5.00E−05	0.0026
ENSOARG00000016878	SYNPO2	32.45	11.47	2.83	5.00E−05	0.0026
ENSOARG00000005941	TNC	8.94	1.57	5.69	5.00E−05	0.0026
ENSOARG00000009529	TGFB1I1	27.59	13.03	2.12	5.00E−05	0.0026
ENSOARG00000011328	VIM	492.83	199.65	2.47	1.50E−04	0.0064
ENSOARG00000018034	ZYX	105.4	43.55	2.42	5.00E−05	0.0026

**Table 4:** **The genes related to the extracellular matrix (ECM)-receptor interaction pathway significantly enriched in the resistant line of the** ***Trichostrongylus*** **selection flock (TSF) in response to a primary** ***Haemonchus contortus*** **infection**

Gene_ID	Symbol	TRI_FPKM	TSI_FPKM	Fold_TRI/TSI	*P*_value	FDR
ENSOARG00000018809	CD44	35.67	19.63	1.82	7.50E−04	0.0229
ENSOARG00000016476	COL3A1	123.25	56.84	2.17	2.50E−04	0.0097
ENSOARG00000006115	COL4A1	46.56	20.73	2.25	5.00E−05	0.0026
ENSOARG00000020503	COL4A4	0.76	1.63	0.47	3.50E−04	0.0125
ENSOARG00000019080	COL6A3	16.59	6.94	2.39	5.00E−05	0.0026
ENSOARG00000019329	FN1	26.36	6.52	4.04	5.00E−05	0.0026
ENSOARG00000008248	HSPG2	36.03	17.16	2.1	2.50E−04	0.0097
ENSOARG00000008285	ITGA1	9.86	5.47	1.8	3.00E−04	0.0113
ENSOARG00000005412	ITGA3	32.43	19.1	1.7	8.00E−04	0.024
ENSOARG00000016181	ITGA5	17.19	9.68	1.78	1.05E−03	0.0291
ENSOARG00000000498	ITGA9	3.5	1.84	1.9	1.00E−04	0.0046
ENSOARG00000007694	LAMA3	11.35	5.25	2.16	1.50E−04	0.0064
ENSOARG00000019180	LAMC1	19.33	10.48	1.85	2.50E−04	0.0097
ENSOARG00000002590	SPP1	13.72	6.55	2.09	1.00E−03	0.0281
ENSOARG00000005941	TNC	8.94	1.57	5.69	5.00E−05	0.0026
ENSOARG00000020058	THBS1	18.62	5.14	3.62	5.00E−05	0.0026
ENSOARG00000003894	THBS3	5.53	2.9	1.9	1.10E−03	0.0302
ENSOARG00000017490	THBS4	72.12	14.02	5.14	5.00E−05	0.0026
ENSOARG00000008752	VWF	15.23	7.91	1.93	5.00E−04	0.0166

**Figure 4:**
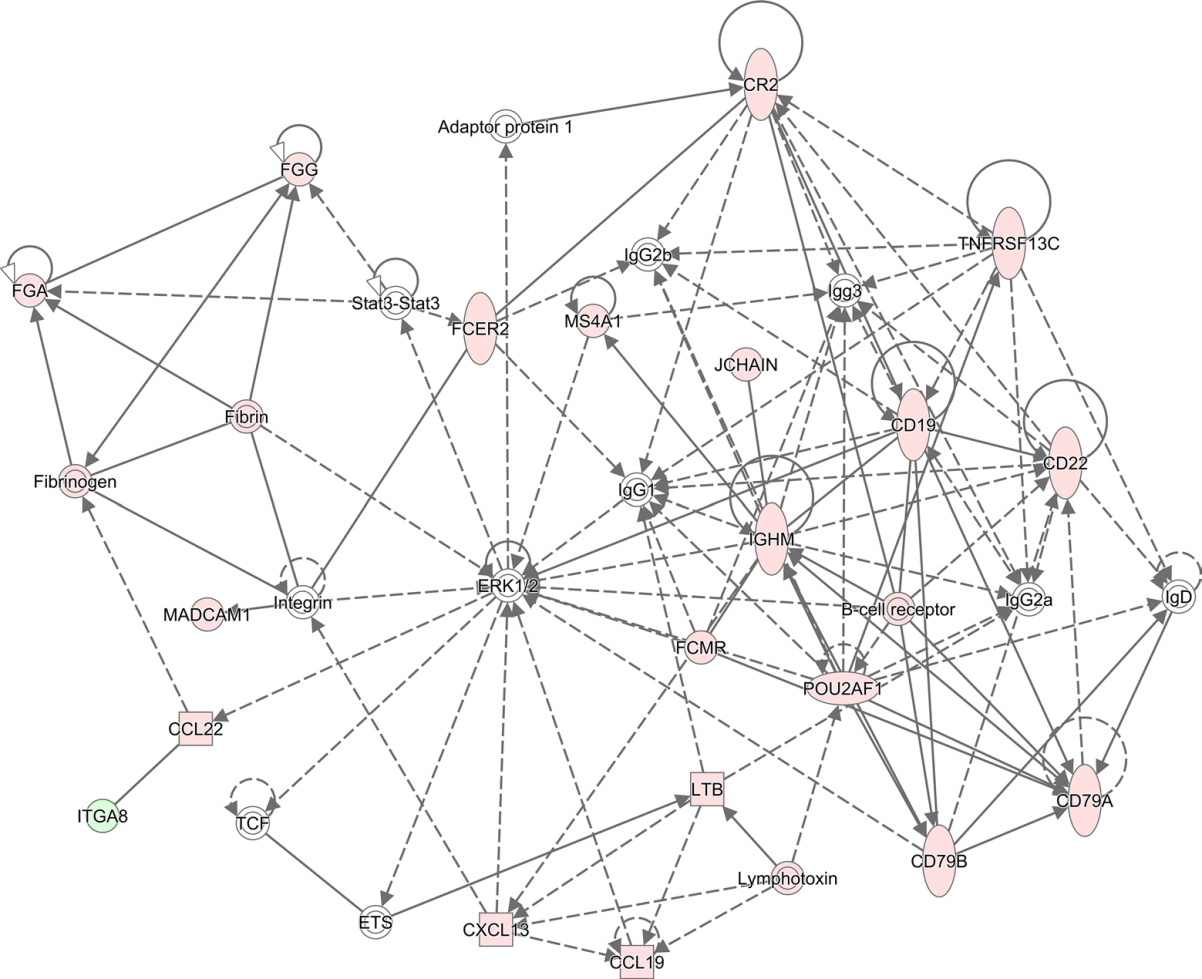
**Visual representation of molecular interactions in the HSF flock.** The molecular interactions for the genes with significantly different abundance between the resistant and susceptible lines in the HSF flock in response to a primary *Haemonchus contortus* infection for 3 days were displayed. The network was inferred using Ingenuity Pathway Analysis (IPA v01-13). Solid lines imply direct relationships between gene products while dotted lines imply indirect interactions. Red: the genes with a significantly higher abundance in the Resistant line than in the susceptible line. Green: the genes with a significantly lower abundance in the Resistant line than in the susceptible line. The degree of changes in gene abundance is represented by the intensity of the color.

**Figure 5:**
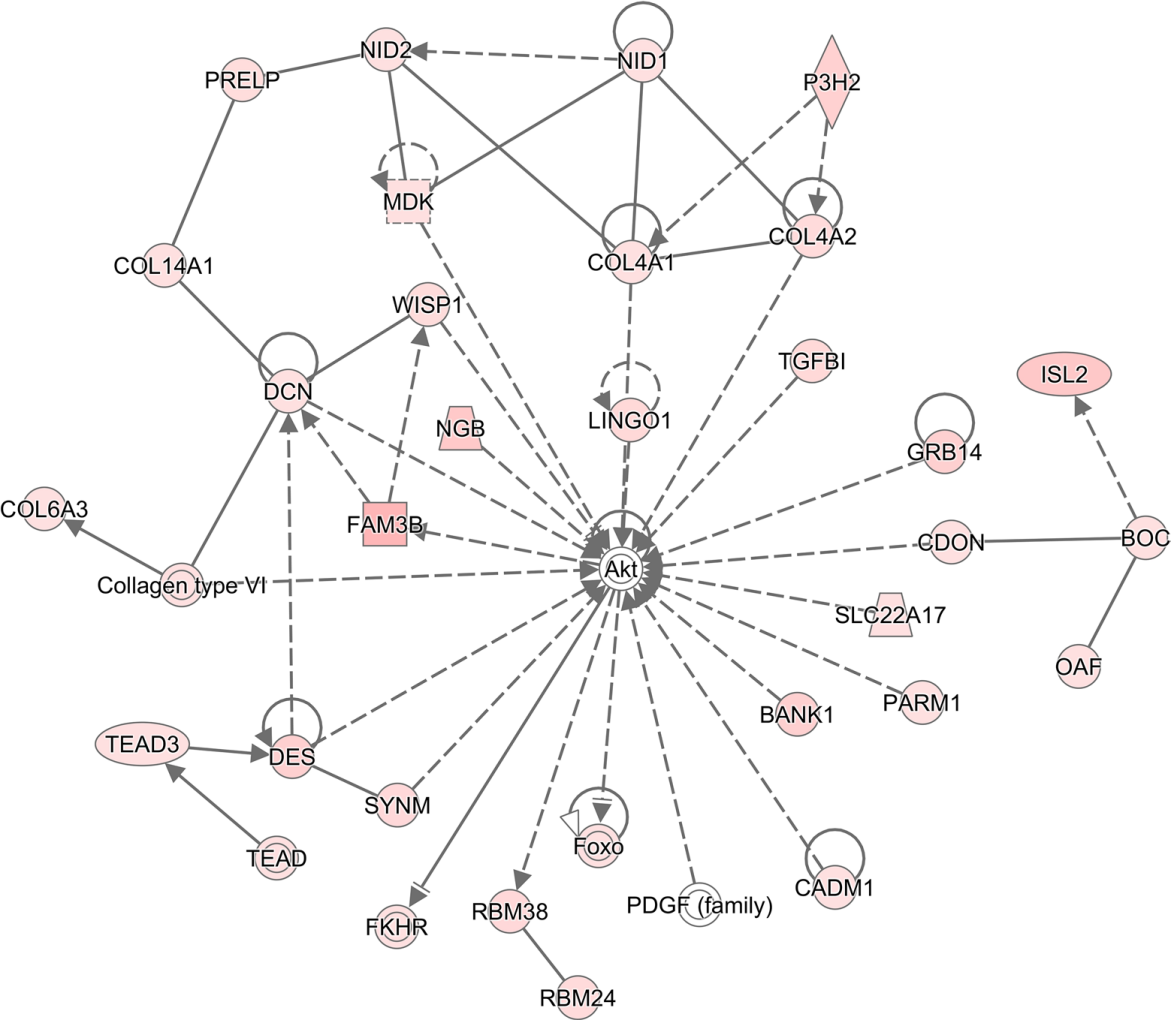
**Visual representation of molecular interactions in the TSF flock.** The network was inferred using Ingenuity Pathway Analysis (IPA v01-13). The genes in the network included those with significantly different abundance between the resistant and susceptible lines in the TSF flock in response to a primary *Haemonchus contortus* infection for 3 days. Solid lines imply direct relationships between gene products while dotted lines imply indirect interactions. Red: the genes with a significantly higher abundance in the Resistant line than in the susceptible line. Green: the genes with a significantly lower abundance in the Resistant line than in the susceptible line. The degree of changes in gene abundance is represented by the intensity of the color.

The resistant lines (R) in the both flocks likely utilized different molecular mechanisms for host resistance to a primary infection, as reflected by the difference in interaction patterns and composition in the inferred molecular networks (Figures 4, 5). In the HSF flock, the resistant line may rely on several B cell related canonical pathways to fight a primary *H. contortus* infection. At least 5 canonical pathways, such as B Cell development, B Cell receptor signaling, B Cell activating factor signaling, PI3K Signaling in B lymphocytes, and altered T Cell and B Cell signaling in arthritis, were significantly enriched in the R line of the HSF flocks. In addition, both agranulocyte and granulocyte adhesion and diapedesis pathways played important roles in this flock. Moreover, it appeared that Complement system as well as coagulation system also played an important role in resistance development in this flock. In contrast, the pathways such as Histamine (and Dopamine) degradation, fatty acid α-oxidation, and acute phase response signaling were seemingly critical in fighting against a primary infection in the TSF flock.

We also analyzed the dataset using the STAR-RSEM-EdegR pipeline. Under the same stringent threshold cutoff (FDR < 0.05), we identified 277 unique genes that displayed a significantly different abundance in one or more comparisons (Additional file 9). Among them, 185 genes, representing approximately 67% of all DEG identified by the STAR-RSEM-EdgeR pipeline, were also detected using the TopHat2–Cufflink–Cuffdiff pipeline. For example, significant changes in the expression of amphiregulin, C–C motif chemokine 25 (CCL25), galectins (LGALS3BP, LGALS7, and LGALS9), granzyme B, prostaglandin G/H synthase 1 (PTGS1 or COX1) and, prostaglandin-endoperoxide Synthase 2 (PTGS2 or COX2) were identified by both pipelines when comparing the TRI group with the TSI group. Moreover, the TopHat2 pipeline also identified transcript isoforms that displayed a significant difference in relative abundance between R and S lines of both flocks in response to the innate infection protocol (Additional file 10). Amphiregulin is a well-known Th2 cytokine enhancing resistance to gastrointestinal nematodes [28]. On the other hand, both pipelines detected a significant upregulation of cadherin 26 (CDH26) only in the resistant line in the HSF flock in response to the innate protocol.

### Real-time RT-PCR confirmation

**Figure 6:**
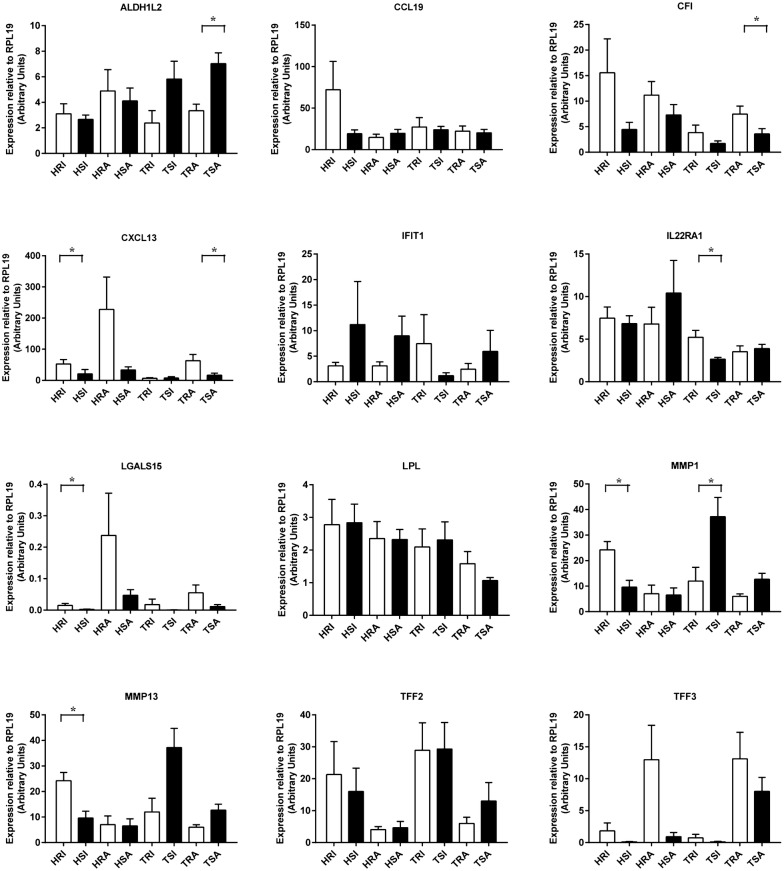
**Real-Time RT-PCR analysis (qPCR) of selected genes.** Relative expression levels calculated from standard curves were normalized to the endogenous control RPL19 gene. Numbers represent mean plus standard error. ALDH1L2: aldehyde dehydrogenase 1 family member L2; CCL19: C–C motif chemokine ligand 19; CFI: complement factor I; CXCL13: C–X–C motif chemokine ligand 13; IFIT1: interferon induced protein with tetratricopeptide repeats 1; IL22RA1: interleukin 22 receptor subunit alpha 1; LGALS15: lectin, galactoside-binding, soluble, 15; LPL: lipoprotein lipase; MMP1: matrix metallopeptidase 1; MMP13: matrix metallopeptidase 13; TFF2: trefoil factor 2; TFF3: trefoil factor 3. *P < 0.05; **P < 0.01.

**Figure 7:**
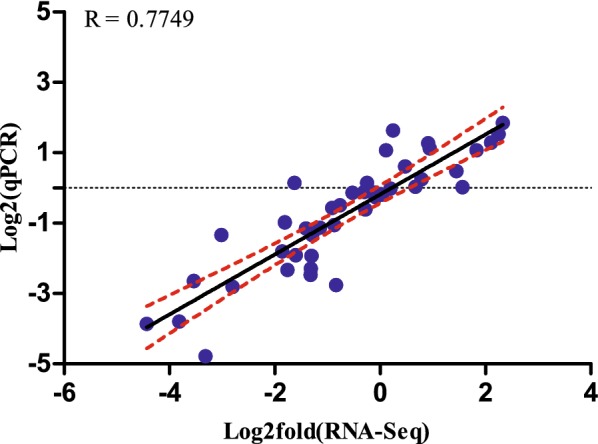
**Linear regression analysis of fold changes calculated from qPCR and RNAseq analysis**. Blue dots represent log2 transformed fold change values of a single gene in an infected sample obtained from qPCR (X-axis) and RNAseq analysis (Y-axis). Dashed lines: 99% confidence interval. R: correlation coefficient.

The RNA-seq results of selected genes were validated in the same sample set by real-time (q) RT-PCR (Figure 6). A correlation coefficient (R) of log2 transformed fold change values between qRT-PCR and RNA-seq platforms was 0.7749 (Figure 7). The expression results of CFI, CXCL13, IFIT1, IL22RA1, LGALS15, LPL, MMP1, and MMP13 obtained using qRT-PCR were in good agreement with the RNA-seq results.

## Discussion

It has long been recognized that differences in host resistance and susceptibility to parasitic infection exist in various sheep breeds [7, 29] and that genetics may play an important role in regulating host resistance, which has spurred efforts to control parasitic infection through selective breeding for naturally resistant sheep. Over 40 years, two flocks, HSF and TSF, each containing a resistant and a susceptible line, were established by selective breeding [9, 16, 18]. The two flocks have been shown to be ideal models for elucidating the molecular mechanisms of immune resistance and susceptibility of sheep to gastrointestinal nematodes [18].

In this study, the number of genes differentially expressed between resistant and susceptible lines in the HSF in response to both primary and tertiary *Haemonchus* infections was significantly fewer than those in the TSF (Figure 2). This may support a notion that long-term selective breeding has an effective impact, at least in part, on the immune response of Merino sheep to *H. contortus* infection. Moreover, our findings provided evidence that different mediators and pathways underlying resistance and susceptibility existed in the two selection flocks, likely due to the difference in their genetic backgrounds. Furthermore, it appeared that the abomasal transcriptome was more responsive to a primary infection than a repeated infection, regardless of the selection flock, as evidenced by a significantly higher number of DEG identified between the R and S lines in response to the innate infection protocol than the acquired infection in both flocks. Following a primary infection, 127 and 726 genes were identified as differentially expressed in the HSF and TSF flocks, respectively. Of these, 38 were shared in both flocks in response to a primary infection, whereas none were identified in both flocks after the tertiary challenge. A proportion of the shared genes differentially expressed following a primary infection have previously been associated with helminth infection. These genes, such as CFI, IFIT1, IFIT2, and LGALS15, may underlie the basis of resistance. CFI is the major complement inhibitor that degrades the activated C3b and C4b in the presence of specific cofactors. CFI deficiency results in secondary complement C3 deficiency due to uncontrolled spontaneous alternative pathway activation, and CFI-deficient individuals suffer from recurring infections [30, 31]. CFI had significantly more abundant transcripts in the abomasum of resistant sheep, which is also observed in the resistant Canaria Hair breed [20]. LGALS15 has been shown to be absent from the normal gastrointestinal epithelium in sheep but induced and secreted into the mucus by epithelial cells within three days after a primary infection with *H. contortus* [32]. The mRNA levels of LGALS15 were significantly up-regulated in the abomasum of resistant sheep in both flocks after a primary infection in this study. In addition, following a primary infection, the LGALS3 gene in the HSF flock and the LGALS3BP gene in the TSF flock were significantly induced in the abomasum of resistant sheep. IFIT genes, induced by host innate immune defenses after pathogen infection, have been suggested to function as antiviral effectors and immunomodulators [33]. IFIT proteins inhibits viral infections by suppressing translation initiation, binding uncapped or incompletely capped viral RNA, sequestering viral proteins or RNA in the cytoplasm, and regulating cell-intrinsic and cell-extrinsic immune responses through pathways that remain to be defined and/or corroborated. Interestingly, following a primary infection, IFIT1 and IFIT2 genes were repressed more in the resistant animals than in the susceptible animals in the HSF flock, while all members of IFIT family (IFIT1, IFIT2, IFIT3, and IFIT5) were significantly upregulated in the resistant line compared to the susceptible line in the TSF flock (Table 2). The divergent pattern of IFIT expression may contribute to different mechanisms utilized by different selection flocks in the acquisition of immunity to *H. contortus* infection. IFIT proteins have not been previously associated with the development of immune responses to parasitic nematode infections. Further study is required to determine the expression profiles in different breeds and uncover their functions in the immune response to nematode infection.

Molecular interactions inferred using IPA provided further evidence that the mechanisms responsible for the development of host resistance in different flocks in response to two different infection protocols differed. The resistant lines in both flocks displayed a stronger and more complex interaction network in response to the innate infection protocol than the acquired protocol. The largest molecular network (Figure 4) in the comparison between HRI and HSI consisted of 20 focus molecules while the largest network in the TRI vs TSI included 29 focus molecules (Figure 5). The major molecular function associated with the former network was humoral immune response and immunological disease while the latter network included organismal injury and abnormalities. Indeed, in the HSF flock in response to the innate infection protocol, J chain and immunoglobulin heavy constant mu (IGHM) seemingly played an important role to the coherence of this network. Mucosal IgA is critical to host immune responses and the development of resistance to *Haemonchus* and *Teladorsagia* infections in sheep. It is negatively correlated with adult worm length and fecundity in a resistant sheep breed [34]. J chain regulates the formation of polymeric IgA and IgM and has a high affinity for the polymeric Ig receptor (PIGR), which may be involved in the development of parasite resistance in cattle [35]. In the bovine abomasum, PIGR, Complement C3, and J chain are among the most abundant transcripts [19]. Together, interactions among multiple molecules consisting of this network, such as CD19, CD 22, and CD79A/B, and chemokines including CCL19, CCL22, and CXCL13 may contribute to enhanced immune responses in the resistant line in the HSF flock to the innate infection. In the latter network (Figure 5), protein kinase B (also known as Akt) occupied a focal position in the network. In contrast to the compositional difference in both networks in response the innate infection protocol, in both flocks in response to the acquired infection protocol, cell cycle, cell death and survival, and cell signaling were among the primary functions in the networks identified (data not shown).

There were seemingly strong relationships between immune responses to parasite infections and the regulation of focal adhesion, extracellular matrix remodeling, and immune cell trafficking, which are involved in the reorganization of the epithelial layer and wound repair and play important roles in gut physiology [36]. Indeed, the rate of epithelial turnover has been shown to be associated with accelerated worm expulsion [16, 17, 37].

Intriguingly, approximately 20% of the differentially expressed genes after a primary infection in the resistant sheep in both flocks were extracellular exosome-related (Additional file 7). The majority of these exosome related genes were significantly upregulated in resistant sheep. Host-derived extracellular exosomes have been shown to be used as defense mechanisms and play important roles in antigen presentation during parasitic infection. For example, intestinal epithelial cells increased the release of antimicrobial peptide-containing exosomes in response to *Cryptosporidium* infection, which is driven by enhanced toll-like receptor 4 signaling following recognition of the protozoan parasite [38]. *Mycobacterium tuberculosis* is also able to induce exosome release from infected macrophages, which consequently promotes recruitment of lymphocytes through heightened proinflammatory chemokine secretion [39]. *Mycobacterium bovis*-infected macrophage-derived exosomes can promote dendritic cell activation and generate antibacterial T cells in vivo [40]. Importantly, vaccination of chickens with *Eimeria* parasite antigen-loaded dendritic cell exosomes has been shown to ameliorate symptoms of avian coccidiosis [41]. However, molecular mechanisms of extracellular exosomes in the development of host resistance to *H. contortus* infection in sheep, especially their roles in eosinophilia and mucosal mastocytosis, warrants further investigation.

## Additional files

**Additional file 1.** **Primer sequences used for qPCR validation.**

**Additional file 2.** **Principal component analysis (PCA) by flocks (A) and by lines (B).**

**Additional file 3.** **Differentially expressed genes (DEGs) between the Resistant and Susceptible lines in the TSF flock.** Genes with significantly different abundance between the Resistant and Susceptible lines in response to a primary *Haemonchus contortus* infection in the *Trichostrongylus* Selection Flock (TSF) at a False Discovery Rate (FDR) < 0.05%.

**Additional file 4.** **DEGs between the Resistant and Susceptible lines in the HSF flock.** Genes with significantly different abundance between the Resistant and Susceptible lines in response to a primary *Haemonchus contortus* infection in the *Haemonchus* Selection Flock (HSF) at a False Discovery Rate (FDR) < 0.05%.

**Additional file 5.** **DEGs between the two lines in the HSF flock in response to a tertiary infection.** Genes with significantly different abundance between the Resistant and Susceptible lines in response to a *Haemonchus contortus* tertiary infection in the *Haemonchus* Selection Flock (HSF) at a False Discovery Rate (FDR) < 0.05%.

**Additional file 6.** **DEGs between the two lines in the TSF flock in response to a tertiary infection.** Genes with significantly different abundance between the Resistant and Susceptible lines in response to a *Haemonchus contortus* tertiary infection in the *Trichostrongylus* Selection Flock (TSF) at a False Discovery Rate (FDR) < 0.05%.

**Additional file 7.** **Extracellular Exosome related genes significantly enriched in both flocks in response to** ***Haemonchus contortus*** **infections.**

**Additional file 8.** **Significantly enriched Database for Annotation, Visualization, and Integrated Discovery (DAVID) terms.** DAVID terms at a False Discovery Rate (FDR) < 10% in the *Trichostrongylus* Selection Flock (TSF) in response to a primary *Haemonchus contortus* infection were shown.

**Additional file 9.** **277 unique differentially expressed genes detected using the STAR-EdgeR pipeline.** 185 of the 277 genes, approximately, 67% of the all DEGs identified by the STAR pipeline are also detected using the same stringency cutoff by the Tophat2-Cufflink-Cuffdiff pipeline.

**Additional file 10.** **Significant transcript isoforms between resistant and susceptible lines in response to** ***Haemonchus contortus*** **infection.** These isoforms were detected using the TopHat2-Cuffdiff pipeline.
