# Supplementary figures and images for: Transcriptome analysis unraveled potential mechanisms of resistance to Haemonchus contortus infection in Merino sheep populations bred for parasite resistance
Source: Vet Res. 2019 Jan 24;50:7. doi: 10.1186/s13567-019-0622-6 (PMC6345051; doi:10.1186/s13567-019-0622-6)

**Additional file 2**

**A**


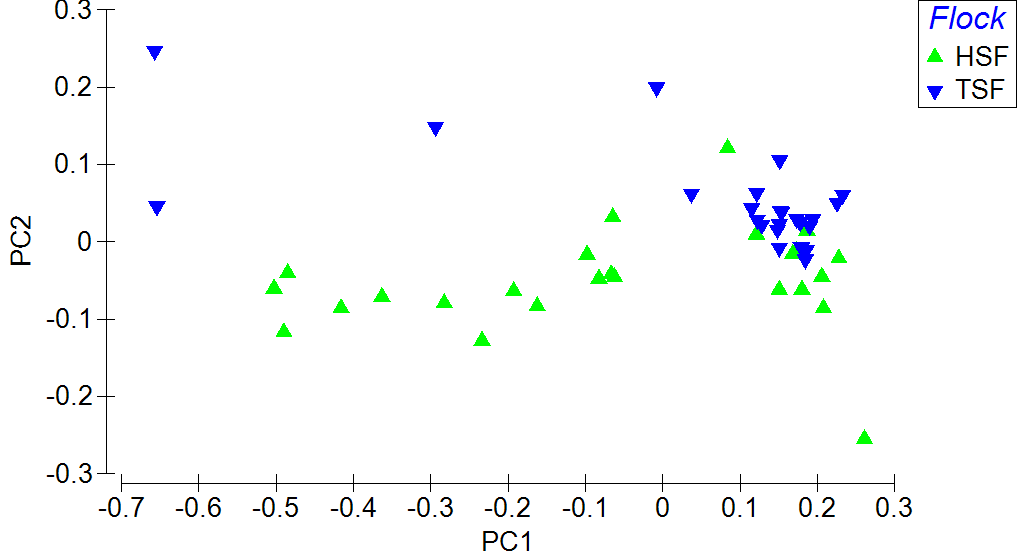


**B**


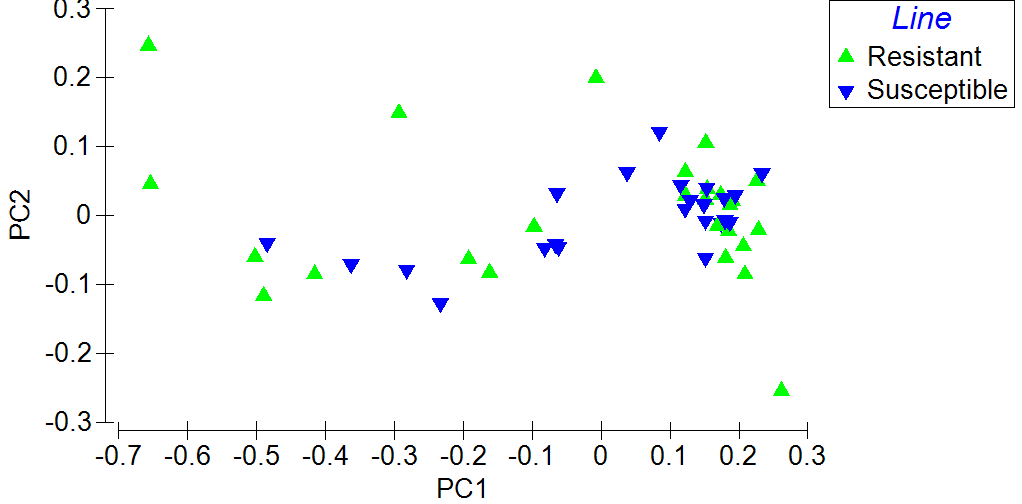

Supplement: Supplementary file 2 — Additional file 2. Principal component analysis (PCA) by flocks (A) and by lines (B). [file 13567_2019_622_MOESM2_ESM.docx]
